# Emerging and Newly Approved Therapies for Antihistamine-Refractory Chronic Spontaneous Urticaria: A Narrative Review

**DOI:** 10.3390/diseases14080276

**Published:** 2026-07-31

**Authors:** Raghad Saeed Asiri, Khaled Abdulwahab Amer, Leen Abdulmohsin Sarhan, Najla Ahmad Jahash, Riham Hamoud Alharbi

**Affiliations:** 1 Department of Internal Medicine, Aseer Central Hospital, Aseer Health Cluster, Abha 62523, Saudi Arabia; 2ragahd2@gmail.com (R.S.A.); najlajahash@gmail.com (N.A.J.); 2 Department of Immunology, Aseer Central Hospital, Aseer Health Cluster, Abha 62523, Saudi Arabia; leen-sarhan@hotmail.com; 3 Department of Pharmacy, Hottat Bani Tamim General Hospital, Hotat Bani Tamim 17981, Saudi Arabia; rihamha@moh.gov.sa

**Keywords:** chronic spontaneous urticaria, omalizumab, dupilumab, remibrutinib, Bruton tyrosine kinase inhibitor, barzolvolimab, KIT, biologic therapy, mast cell

## Abstract

Chronic spontaneous urticaria (CSU) is a mast cell-driven disorder defined by recurrent wheals, angioedema, or both, persisting for longer than six weeks without an identifiable external trigger. Second-generation H1-antihistamines remain first-line therapy, yet a large share of patients stay symptomatic after the dose is raised up to fourfold, and for more than a decade, omalizumab was the only targeted option for those who failed antihistamines. This began to change in 2025, when the interleukin-4 receptor alpha (IL-4Rα) antagonist dupilumab and the oral, covalent Bruton tyrosine kinase (BTK) inhibitor remibrutinib were approved for antihistamine-refractory CSU within months of one another, while the anti-KIT monoclonal antibody barzolvolimab, which depletes mast cells, advanced into the largest phase 3 program the disease has seen. This narrative review outlines the guideline framework that still anchors CSU care, appraises the pivotal efficacy and safety data for omalizumab, dupilumab, remibrutinib, and barzolvolimab, and considers how these mechanistically distinct agents might be positioned and sequenced. The widening choice makes individualized, mechanism-informed treatment a realistic goal, but it also sharpens unresolved questions about patient selection, optimal treatment duration in a naturally remitting disease, and the long-term safety of newer oral and mast cell-depleting approaches.

## 1. Introduction

Chronic spontaneous urticaria ranks among the most common and burdensome conditions seen in allergy and dermatology clinics. It presents with itchy wheals, angioedema, or both, lasting longer than six weeks in the absence of a specific external trigger [1,2,3]. Pooled epidemiological data place the point prevalence of chronic urticaria at roughly one percent of the general population, and the disease tends to run for years rather than weeks, associated with substantial impairment in sleep, mood, work, and overall quality of life [4,5].

For most of the past two decades the treatment ladder was short. Antihistamines came first; omalizumab and, in some settings, ciclosporin were held in reserve for patients who failed them. The difficulty was that this approach left many patients behind. Roughly six in ten adults remain symptomatic on antihistamines even at high doses, and a meaningful subgroup (roughly a quarter to a third) derives little benefit from omalizumab [6,7,8]. Until recently those patients had few evidence-based options left to try.

The picture began to shift in 2025. Dupilumab became the first new targeted therapy approved for CSU in more than a decade, and within months remibrutinib followed as the first oral, mechanism-targeted agent for the disease. In parallel, the mast cell-depleting antibody barzolvolimab reported positive phase 2 data and advanced into large confirmatory trials. Most existing reviews either predate the 2025 approvals or consider these agents individually. Here, we appraise all four mechanisms together in light of the 2025–2026 regulatory and trial developments, and frame the clinical task as one of positioning and sequencing rather than a drug-by-drug catalog. A central theme running through this review is that CSU is not a single disease but a biologically heterogeneous condition, with distinct patterns of mast cell activation across patients; this heterogeneity is the rationale for mechanism-based therapy and for the individualized positioning we propose.

### 1.1. Literature Search and Selection

This is a narrative (non-systematic) review. We searched PubMed/MEDLINE and the ClinicalTrials.gov registry from January 2013 to June 2026; the start point was chosen to coincide with the pivotal omalizumab registration trials, which mark the beginning of the modern targeted era in CSU. Search terms combined disease descriptors (“chronic spontaneous urticaria,” “chronic idiopathic urticaria,” “chronic urticaria,” “antihistamine-refractory urticaria”) with the names of individual agents and their molecular targets (“omalizumab,” “dupilumab,” “remibrutinib,” “Bruton tyrosine kinase,” “BTK inhibitor,” “barzolvolimab,” “anti-KIT”) and with outcome and study-design descriptors (“UAS7,” “urticaria activity score,” “randomized controlled trial,” “phase 2,” “phase 3,” “network meta-analysis,” “guideline”). Reference lists of key trials and reviews were hand-searched for additional sources.

We prioritized peer-reviewed phase 2 and phase 3 randomized controlled trials, their pooled and long-term extension analyses, systematic reviews and meta-analyses, and the current international management guideline. Narrative reviews, mechanistic studies, and consensus statements were included where they added pathophysiological or treatment-positioning context. Inclusion was restricted to English-language reports of studies in humans. We excluded studies of chronic inducible urticaria, isolated case reports, and conference abstracts that had been superseded by full publications. Because several of the agents reviewed were approved or first reported only in 2025–2026, the search was supplemented by regulatory labeling, manufacturer press releases, and recent congress presentations to capture data not yet available as full peer-reviewed articles; these non–peer-reviewed sources are identified as such where they are cited. Given the narrative design, no formal risk-of-bias appraisal or quantitative pooling was undertaken, and the cross-trial comparisons presented below should be read as descriptive rather than as a formal indirect-treatment-comparison analysis. In the interest of transparency, we note the following procedural details. Screening and study selection were performed by two authors independently, with disagreements resolved by discussion and, where necessary, adjudication by a third author. The searches described above returned on the order of several hundred records; after screening of titles and abstracts and removal of duplicates and clearly irrelevant items, roughly one hundred full-text sources were assessed, of which the references cited here were retained as most relevant to the four mechanisms under discussion. No numerical response threshold or additional predefined eligibility criteria were applied beyond those stated above; consistent with the narrative design, selection prioritized relevance to therapeutic mechanism and positioning rather than exhaustive capture, and no formal PRISMA flow or quantitative synthesis was undertaken.

### 1.2. Use of Non–Peer-Reviewed Sources

Because dupilumab’s pediatric extension, the US approval of remibrutinib, and much of the barzolvolimab evidence emerged in 2025–2026, a small number of statements in this review rest on sources that have not undergone full peer review, and we identify them explicitly here so that readers can weigh them accordingly. Specifically, the April 2025 US approval of dupilumab for patients aged ≥12 years and the April 2026 extension to children aged ≥2 years are supported by manufacturer press releases [9,10]; the September 2025 US approval of remibrutinib and its dosing are drawn from the prescribing information [11]; the 52-week remibrutinib extension data [12] and the barzolvolimab off-treatment and dose-finding results [13,14] were, at the time of writing, available as in-press articles and congress presentations; and the sample sizes and status of the phase 3 EMBARQ-CSU trials derive from ClinicalTrials.gov registrations [15,16]. These non-peer-reviewed sources carry a greater risk of incomplete reporting and of revision before final publication, and the corresponding statements should be regarded as provisional. Where full peer-reviewed publications become available, they should supersede the interim sources cited here.

## 2. Disease Mechanism in Brief

CSU is fundamentally a mast cell disorder. Activation of cutaneous mast cells releases histamine, platelet-activating factor, and a range of cytokines, producing the wheal-and-flare reaction and angioedema that define the disease [2,3,17]. Two broad and non-exclusive routes to mastcell activation are recognized. In the type I (autoallergic) pattern, immunoglobulin E (IgE) is directed against autoantigens; in the type IIb (autoimmune) pattern, IgG autoantibodies target IgE itself or its high-affinity receptor, FcεRI. Signaling through FcεRI converges on intracellular Bruton tyrosine kinase, while stem cell factor acting through the KIT receptor sustains mast cell survival and number [18,19]. Beyond the classical IgE/FcεRI axis, additional activating pathways are increasingly recognized, including MRGPRX2-mediated (non-IgE) mast cell activation by endogenous and exogenous secretagogues, complement-derived anaphylatoxins such as C5a, and neuroimmune interactions between sensory neurons and mast cells; their relative contribution varies between patients and remains incompletely defined [17,19].

**Figure 1:**
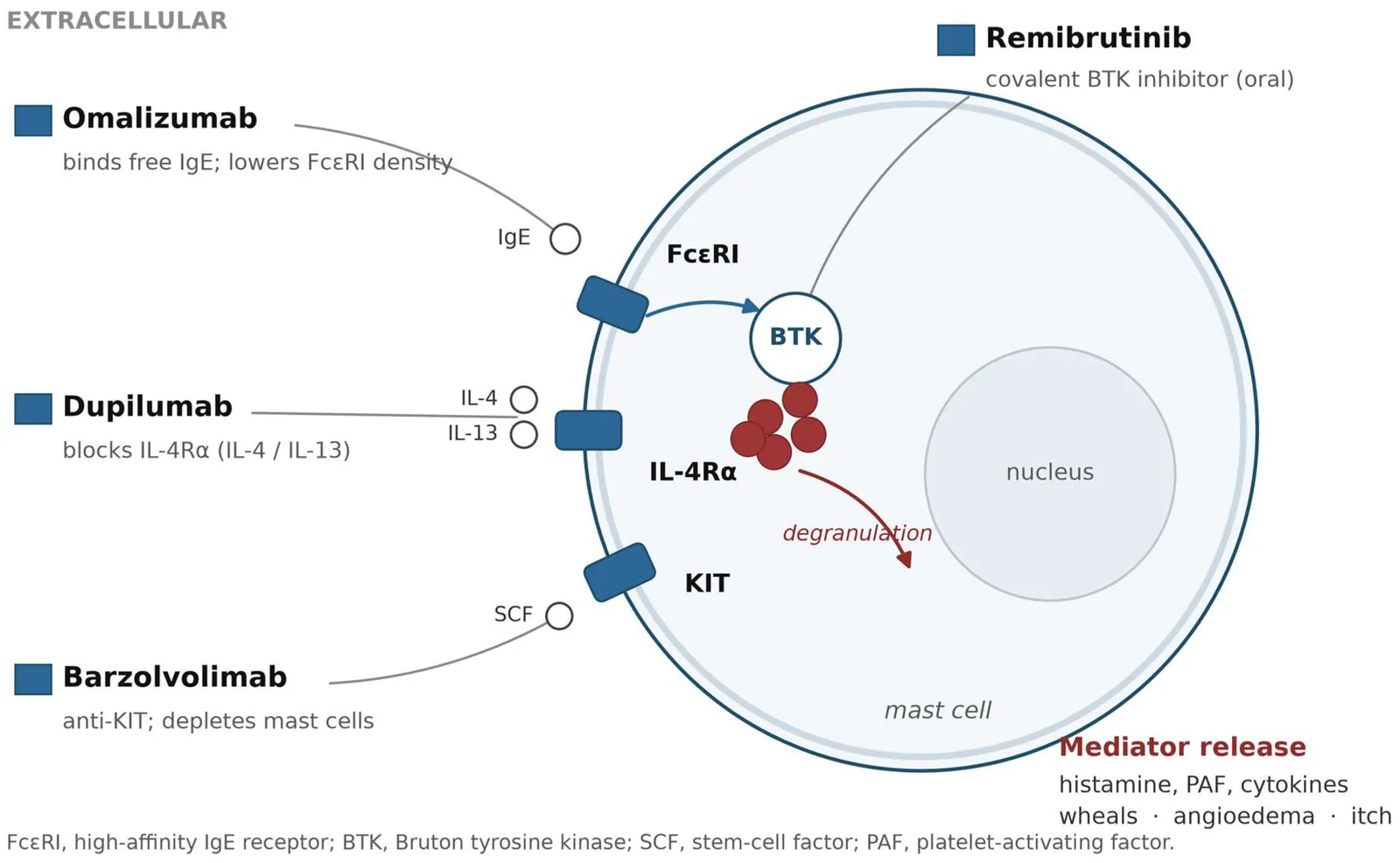
Principal molecular targets in CSU and the sites of action of approved and emerging therapies. Mast cell activation proceeds through the high-affinity IgE receptor (FcεRI) and downstream Bruton tyrosine kinase (BTK), culminating in degranulation and the release of histamine and other mediators; stem cell factor signaling through KIT maintains mast cell survival. Omalizumab binds free IgE and lowers FcεRI density, dupilumab blocks the shared IL-4 receptor alpha subunit, remibrutinib covalently inhibits BTK, and barzolvolimab targets KIT to deplete mast cells. PAF, platelet-activating factor.

This heterogeneity explains why patients respond unevenly to drugs that act at different points along the pathway. An agent that neutralizes IgE, one that blocks type 2 cytokine signaling, one that silences an intracellular kinase, and one that removes the mast cell altogether are not interchangeable; they intervene at distinct nodes, and a patient refractory to one mechanism may still respond to another. Figure 1 maps the principal molecular targets and the agents that act on them.

## 3. Guideline-Based Management

The international EAACI/GA^2^LEN/EuroGuiDerm/APAAACI urticaria guideline remains the dominant framework for treatment [1]. Treatment is structured in steps: a licensed-dose second-generation H1-antihistamine first, followed by up-titration of that same antihistamine (to as much as four times the standard dose) when symptoms persist, and only then the addition of a targeted agent. Omalizumab is recommended as the next step for antihistamine-refractory disease, with ciclosporin reserved for patients who do not respond adequately to it [1,20].

**Figure 2:**
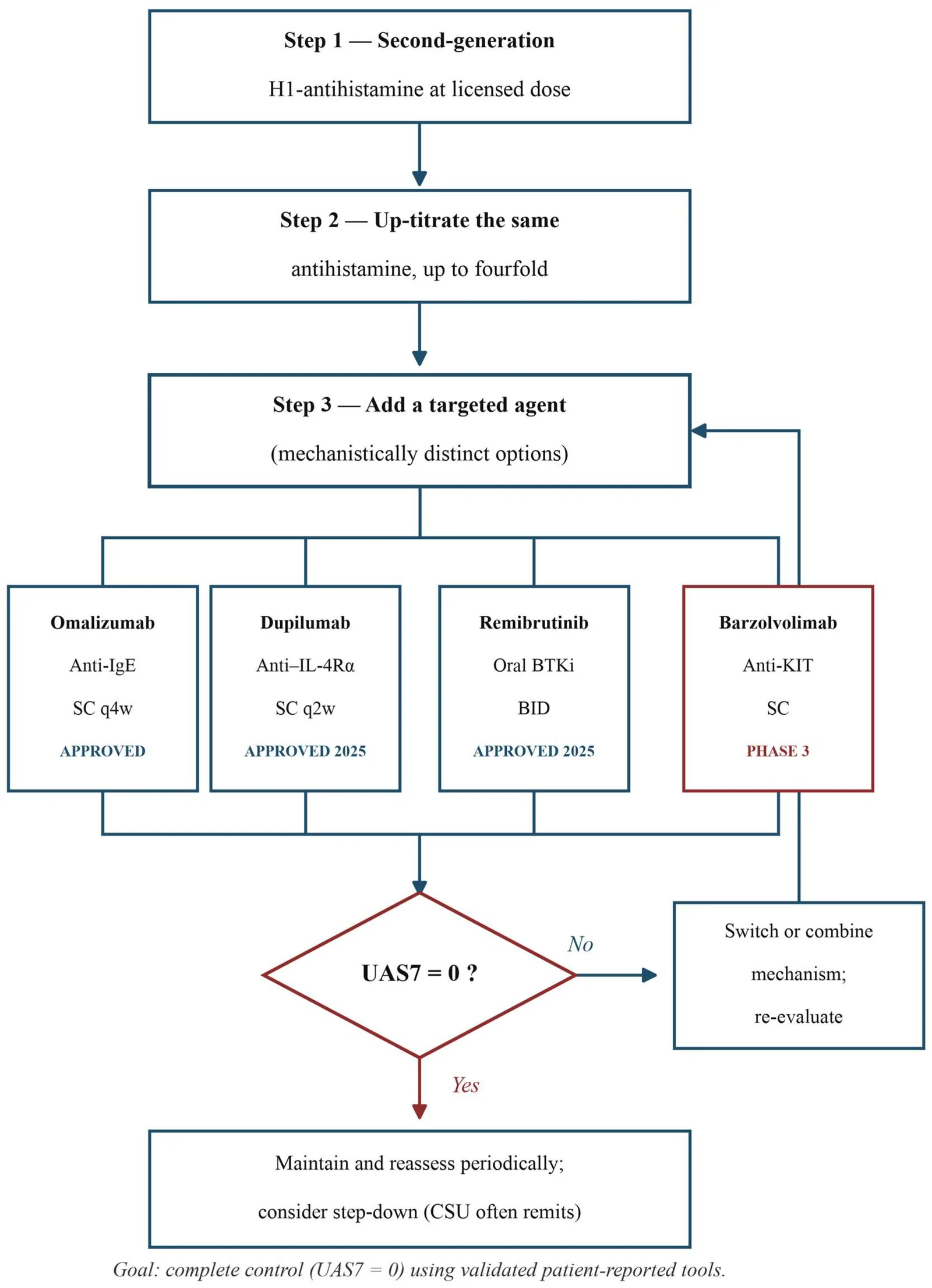
Stepwise management of CSU under the current guideline framework, showing where newly approved and emerging targeted agents fit. Omalizumab remains the established step after antihistamine up-titration; dupilumab and remibrutinib are now approved options at the same step, and barzolvolimab is in phase 3 of development for refractory disease. Treatment is directed toward complete control (UAS7 = 0) and reassessed periodically because the disease often remits. UAS7: weekly Urticaria Activity Score.

Two principles in the guideline matter as much as the sequence itself. The treatment goal is complete symptom control (a weekly Urticaria Activity Score [UAS7] of 0) rather than partial improvement, and disease activity is tracked with validated patient-reported tools such as the UAS7 [21]. Because CSU frequently remits on its own, therapy should also be reassessed at intervals, with step-down or discontinuation considered once control is achieved [22]. The most recent full guideline predates the 2025 approvals, and an update incorporating the agents discussed below is expected in future guideline iterations; in the interim, regulatory labeling and trial evidence have begun to shape how clinicians position these drugs. The current stepwise approach, with the newer agents mapped onto it, is shown in Figure 2. At the time of writing, the 2025–2026 approvals have not yet been formally incorporated into updated national or international guideline documents, and practice differs somewhat between regions: in both the United States and Europe, omalizumab remains the established step after antihistamine up-titration, but the newer approved agents (dupilumab and remibrutinib) are being adopted at a pace that reflects local labeling, reimbursement, and availability rather than a harmonized guideline recommendation.

## 4. Anti-IgE Therapy: Omalizumab

Omalizumab is a recombinant humanized monoclonal antibody that binds the constant (Cε3) domain of free IgE. By lowering circulating free IgE it leads, over weeks, to downregulation of the high-affinity IgE receptor (FcεRI) on mast cells and basophils, reducing the cells’ readiness to degranulate; additional IgE-independent effects on mast cell stability have also been proposed [2,17]. It was approved for antihistamine-refractory CSU in 2014 in both the United States and the European Union and, for more than a decade, was the only targeted option positioned after antihistamine up-titration. It is given subcutaneously every four weeks. Two fixed doses are licensed, 150 mg and 300 mg, and although both are effective, response is dose-dependent and the 300 mg dose is the more effective and the one used in most practices; unlike ciclosporin, it requires no routine laboratory monitoring [1,2].

Efficacy was established in three phase 3, randomized, double-blind, placebo-controlled trials. ASTERIA I and ASTERIA II enrolled patients who remained symptomatic on licensed-dose H1-antihistamines, while GLACIAL studied a more refractory population that had also failed up-to-fourfold antihistamine dosing together with an H2-antihistamine and/or a leukotriene-receptor antagonist [23,24,25]. At the 300 mg dose, complete response (UAS7 = 0) at week 12 was reached by 35.8% of patients in ASTERIA I, 44.3% in ASTERIA II, and 33.7% in GLACIAL, versus 8.8%, 5.1%, and 4.8% on placebo, respectively; well-controlled disease (UAS7 ≤ 6) was correspondingly achieved by roughly half to two-thirds of patients (51.9%, 65.8%, and 52.4%) against placebo rates of 11.3%, 19.0%, and 12.0% [23,24,25]. Responses were dose-dependent and largely reproducible across the three trials. Onset was relatively prompt: some patients improved within the first four weeks, with a median time to well-controlled disease of about six weeks, although complete clearance typically took longer (median roughly twelve weeks) and a subset responded only with continued dosing [23,24,25]. These week-12 complete-response rates should not be compared directly across ASTERIA I, ASTERIA II, and GLACIAL, because the trials differed in baseline disease severity and in inclusion criteria. GLACIAL, in particular, enrolled a more refractory population that had also failed up-to-fourfold antihistamine dosing together with an H2-antihistamine and/or a leukotriene-receptor antagonist.

Omalizumab is generally well tolerated, and across the pooled pivotal trials, the overall rate of adverse events was similar to placebo [23,24,25]. The most frequently reported events are headache, nasopharyngitis and other upper-respiratory infections, arthralgia, and injection-site reactions; the latter are dose-related but infrequent and, in the CSU trials, were not severe [2,8]. The principal safety consideration is a small risk of anaphylaxis, estimated at roughly 0.1–0.2% and recognized chiefly from the drug’s longer use in asthma; it can occur with any dose and is not confined to the first injection, so an appropriate post-injection observation period and access to emergency treatment are advised [2]. A paradoxical worsening of urticaria has been described rarely. Reassuringly, long-term and real-world CSU cohorts followed for several years have not shown an excess of malignancy, thromboembolism, or serious infection, and the agent carries one of the most mature safety records among biologics used in the disease [8].

Its limitations are well recognized and were precisely what motivated the search for alternatives: a sizeable subgroup (roughly a quarter to a third) responds incompletely or not at all, the drug must be given by subcutaneous injection, response can be slow to mature, and a proportion of patients relapse after it is stopped, sometimes requiring retreatment [2,8]. These gaps defined the unmet need that the newer agents set out to address. In practice, several strategies are used for partial or slow responders before the drug is abandoned: a higher likelihood of response is associated with a type I (autoallergic, high-IgE) phenotype whereas type IIb (autoimmune) disease with low IgE and positive basophil tests tends to respond more slowly and less completely; options for incomplete responders include continuing beyond 12 weeks to allow late responses, up-titration to 450–600 mg, or shortening of the dosing interval to every two weeks in some settings (both off-label), and, where these fail, transition to an alternative mechanism [2,8].

## 5. A New Biologic: Dupilumab

Dupilumab is a fully human monoclonal antibody directed against the shared alpha subunit of the interleukin-4 receptor, through which it blocks signaling by both interleukin-4 and interleukin-13, two central drivers of type 2 inflammation [26]. In April 2025, it became the first targeted therapy approved for CSU in over a decade, for patients aged 12 years and older who remain symptomatic on H1-antihistamines; a subsequent decision in April 2026 extended the indication to children as young as 2 years, making it the first biologic available for this younger group [9,10].

**Figure 3:**
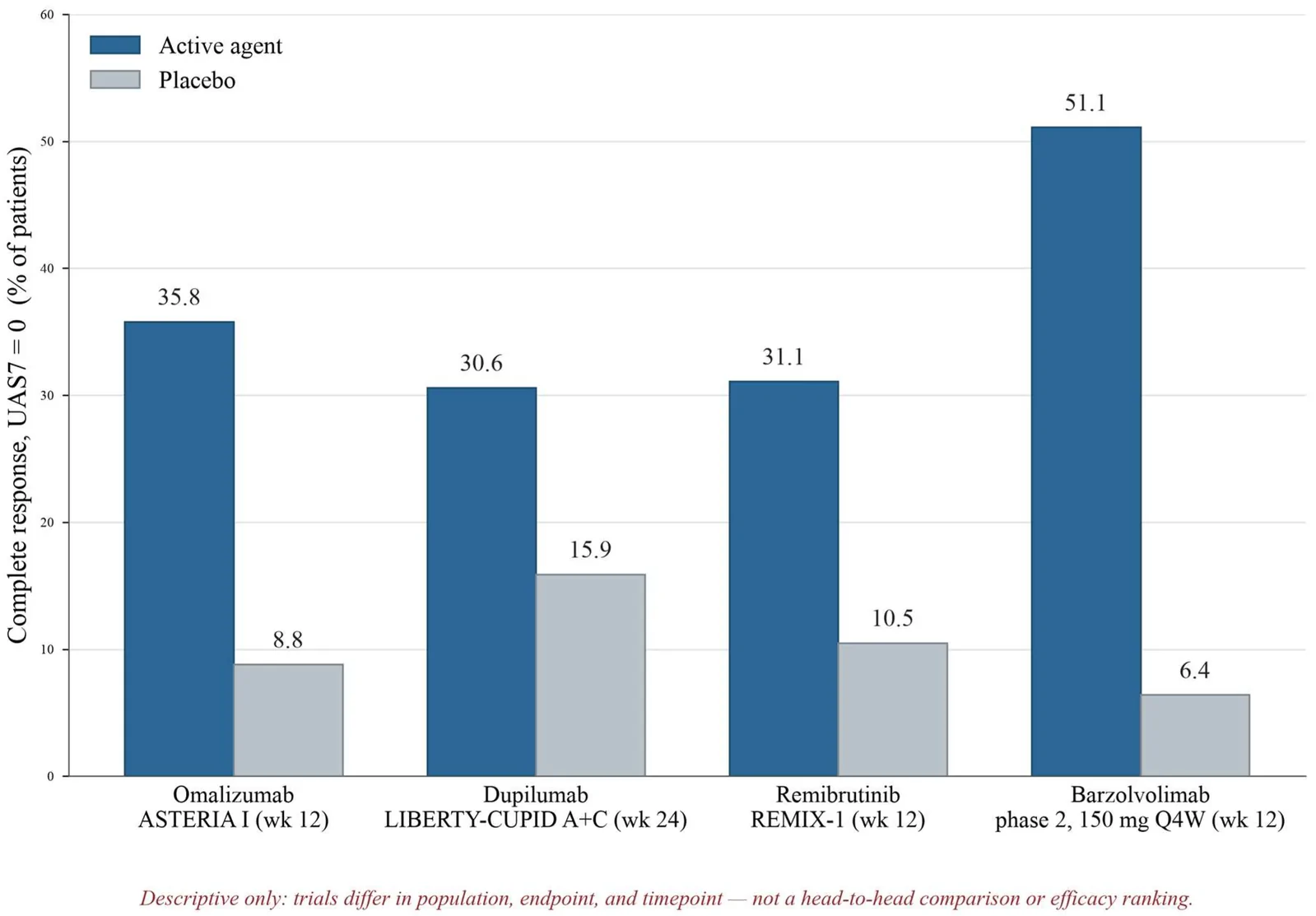
Complete response (UAS7 = 0) reported in the pivotal and phase 2 trials, shown as within-trial active-versus-placebo contrasts. Endpoints were assessed at week 12 (omalizumab [ASTERIA I, 300 mg], remibrutinib [REMIX-1, 25 mg twice daily], barzolvolimab [phase 2, 150 mg every 4 weeks]) or week 24 (dupilumab [pooled LIBERTY-CUPID Studies A and C]). Because the trials differ in population, endpoint, and timepoint, the figure is descriptive and is not a substitute for head-to-head trials or network meta-analysis [23,27,28,29]. UAS7: weekly Urticaria Activity Score. The numerical values shown for different agents must not be interpreted as a comparative efficacy ranking; a caveat to this effect is displayed within the figure itself.

Approval rested on the phase 3 LIBERTY-CUPID program. Studies A and C, conducted in biologic-naive patients, both met their primary and key secondary endpoints, with dupilumab reducing itch severity and urticaria activity significantly more than placebo at week 24 [26,27]. In the pooled Study A and C analysis, the least-squares mean change in UAS7 was −19.3 with dupilumab versus −13.1 with placebo, and significantly more patients reached well-controlled disease (UAS7 ≤ 6: 43.1% vs. 23.4%) and complete response (UAS7 = 0: 30.6% vs. 15.9%) [27]. In other words, beyond the 30.6% who cleared completely, a further one in eight patients reached at least well-controlled disease, for an overall well-controlled rate of 43.1%. Study B, in patients with an inadequate response to or intolerance of omalizumab, provided supportive efficacy and safety data in that harder-to-treat population [26]. The week-24 complete-response rate with dupilumab (30.6%) is numerically lower than the week-12 complete-response figures reported historically with omalizumab in comparable biologic-naive populations (ASTERIA I, 35.8%; ASTERIA II, 44.3%; see Section 4); the populations, endpoints, and assessment timepoints differ, however, so the comparison is indirect and cannot be interpreted as evidence of superiority or inferiority of either agent; these within-trial contrasts against placebo are presented descriptively in Figure 3 rather than as a head-to-head ranking. The safety profile in the CUPID program was consistent with the well-characterized dupilumab class profile: injection-site reactions were the most common events, and conjunctivitis—frequently seen when dupilumab is used in atopic dermatitis—was reported, warranting attention in patients with ocular surface disease. Transient blood eosinophilia can occur early in treatment and is usually asymptomatic and self-limiting, but rare cases of clinically significant hypereosinophilia have been described with dupilumab in other indications. Paradoxical inflammatory reactions, including new or worsening arthralgia and cutaneous eruptions, have also been reported across the dupilumab experience and should be monitored as real-world CSU data accumulate [26,27].

Because dupilumab is effective across a family of type 2 inflammatory diseases (atopic dermatitis, asthma, prurigo nodularis, and others), it is—as a matter of expert opinion rather than of direct comparative evidence, and based on its established efficacy in these other type 2 inflammatory diseases—a reasonable choice for the CSU patient who also carries one of these atopic comorbidities, where a single agent may address more than one problem. Two caveats temper this positioning. First, the onset of dupilumab is comparatively gradual, with pivotal endpoints assessed at week 24, whereas remibrutinib and barzolvolimab show measurable benefit within the first one to two weeks; speed of response may therefore favor the latter agents when rapid control is required. Second, the biological rationale for efficacy in CSU is still incompletely understood, because the precise contribution of IL-4 and IL-13 to mast cell-driven urticaria is less well defined than in other type 2 diseases, and the observed benefit may reflect broader modulation of the type 2 inflammatory milieu rather than a single dominant pathway [26,27].

## 6. Oral BTK Inhibition: Remibrutinib

Remibrutinib is an oral, highly selective, covalent inhibitor of Bruton tyrosine kinase, the enzyme that relays signals downstream of FcεRI in mast cells and basophils. By interrupting that cascade, it suppresses mediator release without removing the cells, combining a targeted mechanism with the convenience of a twice-daily tablet that requires no laboratory monitoring [11,28].

Efficacy was established in the identically designed phase 3 REMIX-1 and REMIX-2 trials, which randomized adults with CSU inadequately controlled on second-generation H1-antihistamines, in a 2:1 ratio, to remibrutinib 25 mg twice daily or placebo over a 24-week double-blind period followed by open-label extension to 52 weeks [12,28]. At the week 12 primary endpoint, remibrutinib produced a markedly greater fall in UAS7 than placebo (least-squares mean change −20.0 vs. −13.8 in REMIX-1 and −19.4 vs. −11.7 in REMIX-2). More patients reached well-controlled disease (UAS7 ≤ 6: 49.8% vs. 24.8% in REMIX-1) and complete response (UAS7 = 0: 31.1% vs. 10.5% in REMIX-1; 27.9% vs. 6.5% in REMIX-2). Improvement appeared within the first weeks and was sustained through 52 weeks, including in patients who crossed over from placebo, and pooled and meta-analytic data confirmed the effect across subgroups [12,30].

The safety profile was favorable overall, with rates of adverse events similar to placebo. The most notable signal was a modest excess of petechiae (3.8% vs. 0.3%), consistent with the known effects of BTK inhibition on platelet function and not associated with broader bleeding concerns in the trials [28]. With its approval by the US Food and Drug Administration in September 2025, remibrutinib became the first BTK inhibitor licensed for CSU and the first oral targeted alternative to injectable biologics [11]. Two practical points deserve mention. Twice-daily oral dosing, while more convenient than injection for many patients, introduces adherence considerations that do not apply to agents given every few weeks. In addition, remibrutinib is one of several BTK inhibitors in development for CSU; rilzabrutinib and other next-generation covalent and reversible BTK inhibitors are at earlier stages, and how these agents will compare in efficacy, selectivity, and long-term safety is not yet known [8]. Several theoretical concerns follow from the mechanism and from experience with earlier BTK inhibitors. First-generation, less selective agents used in haemato-oncology (for example, ibrutinib) have been associated with atrial fibrillation, hypertension, bleeding, and infection, effects attributed partly to off-target kinase inhibition. Remibrutinib is a highly selective covalent BTK inhibitor and the CSU trials did not show these signals, but the exposure to date is short relative to a potentially chronic indication. Longer-term pharmacovigilance for infection (reflecting BTK’s role in immune cell signaling), for cardiovascular events, and for bleeding beyond the observed petechiae will be needed before the safety of sustained BTK inhibition in an otherwise healthy CSU population can be considered established [11,28].

## 7. Mast Cell-Directed Therapy: Barzolvolimab

Barzolvolimab is an investigational humanized monoclonal antibody against KIT, the receptor for stem cell factor. Because KIT signaling is required for mast cell survival, blocking it depletes the very cell that drives CSU [19,29]. In a phase 2 dose-finding trial, 208 patients with antihistamine-refractory disease (UAS7 ≥ 16) were randomized to one of three barzolvolimab regimens or placebo. The agent met its primary endpoint at all doses, and at week 12 complete response (UAS7 = 0), it reached 51.1% with 150 mg every 4 weeks and 37.5% with 300 mg every 8 weeks versus 6.4% with placebo. With continued treatment, complete-response rates rose further, reaching roughly 71% by 52 weeks, and clinical benefit persisted for months after dosing stopped [13,14,29].

Targeting the mast cell itself, rather than one upstream activating signal, gives this approach a theoretical reach across the mechanistic spectrum of CSU, including patients who have failed IgE- or cytokine-directed therapy. The characteristic adverse effects reflect on-target KIT inhibition: reversible lightening of hair color and transient, reversible reductions in blood cell counts, alongside injection-site reactions [29]. Two large phase 3 trials, EMBARQ-CSU1 and EMBARQ-CSU2, together randomized 1939 patients and completed enrollment in early 2026; their results will determine whether the phase 2 promise translates into a durable, well-tolerated option, and will clarify the consequences of sustained mast cell depletion [15,16]. It is important to distinguish what is currently established from what remains preliminary or aspirational: the phase 2 efficacy signal and the characteristic on-target effects are established within that single dose-finding trial; the durability of response, the off-treatment benefit, and the broad applicability across refractory endotypes are preliminary observations that require phase 3 confirmation; and the positioning of barzolvolimab as an option for multi-refractory disease remains a future expectation contingent on the EMBARQ-CSU program. The favorable early data should be read in that light rather than as evidence of established superiority. The longer-term biological consequences of sustained mast cell depletion are not yet known: mast cells contribute to host defense, wound healing, and tissue homeostasis, so the effects of removing them over months to years remain a genuine uncertainty, as does the durability and reversibility of the KIT blockade. The transient reductions in neutrophil counts and the changes in hair and skin pigmentation observed in phase 2 suggest that periodic hematological monitoring and patient counseling about reversible pigmentary changes are likely to be required, and the phase 3 program is expected to characterize these effects more fully [19,29].

The relative performance of these agents on their pivotal endpoints is summarized in Table 1, and the within-trial complete-response data are shown in Figure 3.

## 8. Comparative Positioning and Sequencing

With several targeted options now in hand, treatment is shifting away from a single linear ladder toward a more individualized, mechanism-informed strategy. Direct comparisons remain scarce, so much of the current reasoning rests on indirect evidence. A network meta-analysis of the randomized trials suggests that omalizumab still delivers among the highest complete-control rates of the approved agents, that remibrutinib offers broadly comparable complete-response figures with the practical advantage of oral dosing, and that dupilumab provides a pathway-distinct alternative that may be preferred when atopic comorbidity is present [31,32]. These indirect signals must be interpreted with restraint: remibrutinib should not yet be regarded as an established equivalent of omalizumab, because no head-to-head trial has been reported, indirect comparisons carry the limitations noted above, and the long-term safety record of omalizumab—accumulated over more than a decade of use—remains substantially more mature than that of any of the newer agents. Barzolvolimab, whose efficacy data are so far limited to a single phase 2 trial, could become a valuable option for the most treatment-resistant disease should its phase 3 program confirm the early signal; until then, its durability, its safety beyond one year, and the biological consequences of sustained mast cell depletion remain genuinely uncertain, and the enthusiasm its early data have generated should be tempered accordingly.

These cross-trial estimates must be read with caution, because the studies differ in population, endpoint, and assessment timepoint. Figure 3 therefore presents the within-trial contrast against placebo for each agent rather than implying a head-to-head ranking. In everyday practice, the choice will turn less on small differences in trial numbers than on route of administration and patient preference, the presence of type 2 comorbidities, prior response to or intolerance of omalizumab, the speed of onset required, monitoring demands, and access or cost. Table 2 lays out these practical considerations side by side. The design and population differences that underlie this caution are set out side by side in Table 3, which is intended to be read alongside Figure 3 so that the numerical contrasts are interpreted in the light of the trials’ methodological heterogeneity.

This is a narrative, non-systematic review, and its comparative statements rest largely on indirect, cross-trial evidence; for the newest agents, some data derive from congress presentations and regulatory announcements rather than peer-reviewed head-to-head trials. The positioning suggestions below should be read with that limitation in mind.

### 8.1. Cost, Access, and Real-World Implementation

Beyond trial efficacy, the choice among targeted agents is shaped heavily by cost, reimbursement, and regional availability, and these factors often dominate real-world decisions. The approved biologics and the oral BTK inhibitor are all high-cost therapies whose accessibility varies widely between healthcare systems and payers. In many settings, access to omalizumab is contingent on documented failure of high-dose antihistamines, and access to the newer agents may still be more restricted while reimbursement pathways are established. Route of administration carries its own resource implications: subcutaneous biologics may require clinical visits or arrangements for home administration, whereas an oral agent shifts the burden toward adherence and community prescribing. Patient preference—needle avoidance, dosing frequency, and the acceptability of long-term therapy in a naturally remitting disease—further modulates real-world uptake. A mechanism-informed positioning strategy is therefore only realizable within the constraints of local formularies, and the practical value of the newer agents will depend as much on affordability and access as on their comparative efficacy.

### 8.2. Special Populations

Evidence in special populations remains limited for all four agents, and most guidance is extrapolated rather than trial-derived. In older adults, comorbidity and polypharmacy argue for agents with minimal monitoring burden and well-characterized safety. Omalizumab has the longest track record, while the cardiovascular and bleeding considerations noted for BTK inhibition warrant particular care in this group. In pregnancy and breastfeeding, omalizumab has the most reassuring accumulated human data and is generally considered the preferred targeted option when one is required, whereas human safety data for dupilumab are more limited and those for remibrutinib and barzolvolimab are essentially absent, so the latter agents are best avoided pending further data. In immunosuppressed patients and those with severe comorbidity, the theoretical infection risk of sustained BTK inhibition and of mast cell depletion should be weighed against the more established profile of anti-IgE therapy. Finally, use in children remains confined to regulatory approvals—dupilumab down to age 2 years and, for antihistamine-refractory disease, omalizumab in adolescents—while remibrutinib and barzolvolimab have been studied in adults only; off-label pediatric use of the newer agents is not currently supported by trial evidence.

## 9. Future Directions

More than twenty compounds are in clinical development for CSU, aimed at mast cell activation, the IgE axis, and downstream immune signaling [8]. Several BTK inhibitors beyond remibrutinib and additional mast cell-directed antibodies are advancing, and the first head-to-head trials are now underway; RECLAIM, for example, compares remibrutinib directly with dupilumab at early timepoints, a design that indirect analyses cannot replace [33].

Interest is also growing in inhibition of the Janus kinase (JAK)–tyrosine kinase 2 (TYK2) axis, which lies downstream of multiple cytokine receptors implicated in mast cell and type 2 inflammation. In a phase Ib, randomized, placebo-controlled pilot study, the selective TYK2/JAK1 inhibitor TLL-018 reduced disease activity in patients with antihistamine-refractory CSU, providing early proof of concept for oral JAK-pathway inhibition in this setting [34]. Supportive real-world and case-based evidence has accompanied these trial data: a single-center retrospective study reported outcomes and tolerability of JAK inhibitors across chronic spontaneous and chronic inducible urticaria [35], and a case series described benefits from JAK inhibition in patients refractory to both antihistamines and omalizumab [36]. Experience in other type 2 and pruritic dermatoses—for example, prurigo nodularis, where JAK inhibitors have shown activity in a systematic review of published cases—further supports the biological plausibility of targeting this pathway [37]. These data remain early and are drawn largely from small studies, but JAK/TYK2 inhibition represents a mechanistically distinct oral option that may complement the agents reviewed here, and its role will be clarified by adequately powered randomized trials.

Three questions will shape the field over the next few years. The first is biomarkers. Several candidate markers have been studied as predictors of response or as descriptors of endotype, yet none has entered routine clinical practice. A total IgE level in the low–normal range, together with a positive basophil histamine-release or activation test, IgG anti-thyroid (anti-TPO) antibodies, and low basophil counts, characterizes the type IIb autoimmune endotype, which tends to respond slowly and incompletely to omalizumab; conversely, a high total IgE points toward the type I autoallergic endotype and a more favorable, faster anti-IgE response. Elevated D-dimer reflects coagulation-cascade activation and has been associated with greater disease activity and, in some series, with omalizumab response, while eosinophil signatures may prove relevant to type 2-skewed disease and to agents such as dupilumab. These markers have not been adopted routinely because most derive from small, heterogeneous studies, assays such as the basophil activation test are not standardized or widely available, cut-offs are not validated prospectively, and no biomarker has yet been shown in a randomized design to improve outcomes when used to guide agent selection. Establishing reliable, standardized predictors would let clinicians match patient to mechanism instead of cycling through agents empirically [17]. The second is duration: because CSU often remits, the optimal length of treatment and the safest way to taper or stop remain poorly defined, particularly for the newer oral and depleting therapies [22]. The third is long-term safety, which will only become clear as real-world exposure accumulates, especially for sustained BTK inhibition and mast cell depletion. As these data accumulate, CSU care should move from empirical drug-cycling toward endotype-guided treatment.

## 10. Conclusions

The treatment landscape for antihistamine-refractory CSU has widened substantially. The 2025 approvals of dupilumab and remibrutinib, together with the late-stage advancement of barzolvolimab, have substantially expanded therapeutic options for disease that once depended almost entirely on omalizumab. Clinicians now have agents acting at four distinct points in CSU pathophysiology: IgE neutralization, type 2 cytokine blockade, intracellular signal inhibition, and mast cell depletion. By appraising all four together and treating the clinical decision as one of positioning and sequencing rather than a fixed ladder, this review offers a framework for more individualized, mechanism-informed care. Realizing that benefit will depend on validated tools to match patients to mechanisms and on long-term, ideally head-to-head, evidence to guide how the therapies are sequenced.

## Figures and Tables

**Table 1:** Pivotal evidence for approved and emerging targeted therapies in antihistamine-refractory CSU. Complete-response values are taken from the cited trials and are not directly comparable across agents because populations, endpoints, and timepoints differ. BTK: Bruton tyrosine kinase; FcεRI: high-affinity IgE receptor; IL: interleukin; SC: subcutaneous; UAS7: weekly Urticaria Activity Score; wk: week; y: years.

Agent	Target/Mechanism	Route and Regimen	Pivotal Trial(s)	Complete Response (UAS7 = 0)	Status and Key Safety
Omalizumab	Anti-IgE; lowers free IgE and FcεRI density	SC, every 4 weeks	ASTERIA I/II, GLACIAL (phase 3)	35.8% vs. 8.8% (ASTERIA I, wk 12)	Approved; long real-world safety record
Dupilumab	Anti–IL-4Rα; blocks IL-4 and IL-13	SC, every 2 weeks	LIBERTY-CUPID A, B, C (phase 3)	30.6% vs. 15.9% (pooled A + C, wk 24)	Approved 2025 (≥12 y); 2026 (≥2 y)
Remibrutinib	Oral covalent BTK inhibitor	Oral, 25 mg twice daily	REMIX-1, REMIX-2 (phase 3)	31.1% vs. 10.5% (REMIX-1, wk 12)	Approved Sep 2025; petechiae 3.8% vs. 0.3%
Barzolvolimab	Anti-KIT; depletes mast cells	SC (dose-finding)	Phase 2; EMBARQ-CSU1/2 ongoing	51.1% vs. 6.4% (150 mg Q4W, wk 12)	Investigational; hair-color change, cytopenias

**Table 2:** Practical considerations guiding the selection of targeted therapy in antihistamine-refractory CSU. KIT: stem-cell factor receptor; ph 2: phase 2.

Consideration	Omalizumab	Dupilumab	Remibrutinib	Barzolvolimab
Route	Subcutaneous	Subcutaneous	Oral	Subcutaneous
Typical regimen	Every 4 weeks	Every 2 weeks	Twice daily	Investigational
Onset of effect	Days to weeks	Weeks	Within 1–2 weeks	Within 1–2 weeks (ph 2)
Routine lab monitoring	Not required	Not required	Not required	Blood counts (KIT effect)
Best-suited niche	Established first targeted step	Coexisting type 2 disease	Preference for an oral agent	Multi-refractory disease (if approved)
Status (2026)	Approved	Approved	Approved	Phase 3
Approved age groups	≥12 y (CSU)	≥2 y	Adults (≥18 y)	Investigational (adults studied)
Major contraindications/cautions	Prior anaphylaxis to omalizumab; observation for anaphylaxis	Known hypersensitivity; ocular surface disease (conjunctivitis)	Active bleeding risk; caution with antiplatelet/anticoagulant use	On-target cytopenias and pigmentary change; data limited
Evidence level	Phase 3 + >10 y real-world	Phase 3	Phase 3	Phase 2 (phase 3 ongoing)

**Table 3:** Side-by-side comparison of the design and main characteristics of the pivotal and phase 2 trials of targeted therapies in antihistamine-refractory CSU. The trials differ in population, prior biologic exposure, baseline severity, sample size, primary endpoint, and assessment timepoint; these differences limit cross-trial efficacy comparison, and the complete-response values are therefore not directly comparable across agents. BID: twice daily; H1/H2: histamine-1/-2 receptor; HSS7: weekly Hives Severity Score; ISS7: weekly Itch Severity Score; LTRA: leukotriene-receptor antagonist; ph: phase; UAS7: weekly Urticaria Activity Score; wk: week; y: years.

Trial (Agent; Phase)	Population/Prior Biologic Exposure	Key Inclusion/ Baseline Severity	N-Randomized	Primary Endpoint (Timepoint)	Complete Response, UAS7 = 0 (Active vs. Placebo)	Key Safety Findings
ASTERIA I (omalizumab 300 mg; ph 3)	H1-antihistamine–refractory; biologic-naïve	UAS7 ≥ 16 (moderate–severe)	319	Change in weekly itch severity (wk 12)	35.8% vs. 8.8% (wk 12)	AEs ≈ placebo; headache, nasopharyngitis
ASTERIA II (omalizumab 300 mg; ph 3)	H1-antihistamine–refractory; biologic-naïve	UAS7 ≥ 16 (moderate–severe)	323	Change in weekly itch severity (wk 12)	44.3% vs. 5.1% (wk 12)	AEs ≈ placebo; class profile
GLACIAL (omalizumab 300 mg; ph 3)	Refractory to up-to-4× H1 + H2 and/or LTRA (more refractory); biologic-naïve	UAS7 ≥ 16; broader refractory entry	336	Safety at wk 12 (efficacy secondary)	33.7% vs. 4.8% (wk 12)	AEs ≈ placebo; safety was primary endpoint
LIBERTY-CUPID A (dupilumab; ph 3)	Omalizumab-naïve; H1-refractory; ≥6 y	UAS7 ≥ 16	138	Change in UAS7 or ISS7 (wk 24)	30.6% vs. 15.9% (pooled A + C, wk 24)	Injection-site reactions, conjunctivitis, transient eosinophilia
LIBERTY-CUPID B (dupilumab; ph 3)	Omalizumab-intolerant/incomplete responders (biologic-experienced); ≥12 y	UAS7 ≥ 16	108	Change in UAS7 or ISS7 (wk 24)	Primary endpoint not met; supportive data	Class profile
LIBERTY-CUPID C (dupilumab; ph 3)	Omalizumab-naïve (replicate of Study A); ≥6 y	UAS7 ≥ 16	151	Change in UAS7 or ISS7 (wk 24)	Pooled with Study A (30.6% vs. 15.9%)	Class profile
REMIX-1 (remibrutinib 25 mg BID; ph 3)	Adults ≥ 18 y; inadequately controlled on H1 (2:1 randomization)	UAS7 mean ≈ 30 at baseline	470	Change in UAS7 and ISS7/HSS7 (wk 12)	31.1% vs. 10.5% (wk 12)	Petechiae 3.8% vs. 0.3%; AEs otherwise ≈ placebo
REMIX-2 (remibrutinib 25 mg BID; ph 3)	Adults ≥ 18 y; inadequately controlled on H1 (2:1 randomization)	UAS7 mean ≈ 30 at baseline	455	Change in UAS7 and ISS7/HSS7 (wk 12)	27.9% vs. 6.5% (wk 12)	Petechiae 3.8% vs. 0.3% (pooled)
Barzolvolimab phase 2 (dose-finding)	Antihistamine-refractory; mixed prior biologic exposure	UAS7 ≥ 16	208 (3 regimens + placebo)	Change in UAS7 (wk 12)	51.1% (150 mg Q4W)/37.5% (300 mg Q8W) vs. 6.4% (wk 12)	Hair-color change, transient neutropenia/cytopenias, injection-site reactions

## Data Availability

No new data were created or analyzed in this study. Data sharing is not applicable to this article.

## Abbreviations

The following abbreviations are used in this manuscript:

BTK	Bruton tyrosine kinase
CSU	Chronic spontaneous urticaria
EAACI	European Academy of Allergy and Clinical Immunology
FcεRI	High-affinity IgE receptor
IgE	Immunoglobulin E
IgG	Immunoglobulin G
IL	Interleukin
KIT	Stem cell factor receptor
PAF	Platelet-activating factor
SC	Subcutaneous
SCF	Stem cell factor
UAS7	Weekly Urticaria Activity Score

## References

[1] Zuberbier T., Latiff A.H.A., Abuzakouk M., Aquilina S., Asero R., Baker D., Ballmer-Weber B., Bangert C., Ben-Shoshan M., Bernstein J.A. (2022). The international EAACI/GA2LEN/EuroGuiDerm/APAAACI guideline for the definition, classification, diagnosis, and management of urticaria. Allergy.

[2] Kolkhir P., Bonnekoh H., Metz M., Maurer M. (2024). Chronic spontaneous urticaria: A review. JAMA.

[3] Kolkhir P., Giménez-Arnau A.M., Kulthanan K., Peter J., Metz M., Maurer M. (2022). Urticaria. Nat. Rev. Dis. Primers.

[4] Fricke J., Ávila G., Keller T., Weller K., Lau S., Maurer M., Zuberbier T., Keil T. (2020). Prevalence of chronic urticaria in children and adults across the globe: Systematic review with meta-analysis. Allergy.

[5] Gonçalo M., Gimenéz-Arnau A., Al-Ahmad M., Ben-Shoshan M., Bernstein J., Ensina L., Fomina D., Galvàn C., Godse K., Grattan C. (2021). The global burden of chronic urticaria for the patient and society. Br. J. Dermatol..

[6] Maurer M., Weller K., Bindslev-Jensen C., Giménez-Arnau A., Bousquet P.J., Bousquet J., Canonica G.W., Church M.K., Godse K.V., Grattan C.E.H. (2025). Unmet clinical needs in chronic spontaneous urticaria: A GA2LEN task force report. J. Allergy Clin. Immunol..

[7] Johal K.J., Saini S.S. (2020). Current and emerging treatments for chronic spontaneous urticaria. Ann. Allergy Asthma Immunol..

[8] Kolkhir P., Fok J.S., Kocatürk E., Li P.H., Okas T.-L., Marcelino J., Metz M. (2025). Update on the treatment of chronic spontaneous urticaria. Drugs.

[9] (2025). Dupixent (Dupilumab) Approved in the US as the First New Targeted Therapy in Over a Decade for Chronic Spontaneous Urticaria.

[10] (2026). Dupixent (Dupilumab) Approved in the US as the First Biologic Medicine for Young Children with Uncontrolled Chronic Spontaneous Urticaria.

[11] (2025). Rhapsido (Remibrutinib) Tablets, Prescribing Information.

[12] Giménez-Arnau A.M., Szalewski R., Hide M., Jain V., Khemis A., Lebwohl M., Metz M., Mosnaim G., Palumbo M., Şavk E. (2026). Long-term efficacy and safety of remibrutinib in chronic spontaneous urticaria: 52-week results from the phase 3 REMIX-1 and REMIX-2 studies. J. Allergy Clin. Immunol..

[13] Metz M., Mitha E., Leflein J., Talreja N., Gotua M., Krasowska D., Peter J., Anderson J., Young D., Heath-Chiozzi M. (2026). Randomized dose-finding study of anti-KIT barzolvolimab in patients with chronic spontaneous urticaria. J. Allergy Clin. Immunol..

[14] Chaudhry U., Metz M., Anderson J., Leflein J., Gotua M., Talreja N., Maurer D., Alvarado D., Greenberg S., Paradise E. (2026). Prolonged off-treatment efficacy of barzolvolimab in chronic spontaneous urticaria. J. Allergy Clin. Immunol..

[15] Celldex Therapeutics A Phase 3 Study of Barzolvolimab in Participants with Chronic Spontaneous Urticaria (EMBARQ-CSU1); ClinicalTrials.gov Identifier NCT06445023. NCT06445023.

[16] Celldex Therapeutics A Phase 3 Study of Barzolvolimab in Participants with Chronic Spontaneous Urticaria (EMBARQ-CSU2); ClinicalTrials.gov Identifier NCT06455202. NCT06455202.

[17] Kaplan A., Lebwohl M., Giménez-Arnau A.M., Hide M., Armstrong A.W., Maurer M. (2023). Chronic spontaneous urticaria: Focus on pathophysiology to unlock treatment advances. Allergy.

[18] Kolkhir P., Elieh-Ali-Komi D., Metz M., Siebenhaar F., Maurer M. (2022). Understanding human mast cells: Lessons from therapies for allergic and non-allergic diseases. Nat. Rev. Immunol..

[19] Alvarado D., Maurer M., Gedrich R., Seibel S.B., Murphy M.B., Crew L., Goldstein J., Crocker A., Vitale L.A., Morani P.A. (2022). Anti-KIT monoclonal antibody CDX-0159 induces profound and durable mast cell suppression in a healthy volunteer study. Allergy.

[20] Maíz-Saenz L., Giménez-Arnau A.M. (2022). Chronic spontaneous urticaria guidelines: What is new?. J. Allergy Clin. Immunol..

[21] Hawro T., Ohanyan T., Schoepke N., Metz M., Peveling-Oberhag A., Staubach P., Maurer M., Weller K. (2018). The urticaria activity score—Validity, reliability, and responsiveness. J. Allergy Clin. Immunol. Pract..

[22] Terhorst-Molawi D., Fox L., Siebenhaar F., Metz M., Maurer M. (2023). Stepping down treatment in chronic spontaneous urticaria: What we know and what we don’t know. Am. J. Clin. Dermatol..

[23] Maurer M., Rosén K., Hsieh H.-J., Saini S., Grattan C., Gimenéz-Arnau A., Agarwal S., Doyle R., Canvin J., Kaplan A. (2013). Omalizumab for the treatment of chronic idiopathic or spontaneous urticaria. N. Engl. J. Med..

[24] Saini S.S., Bindslev-Jensen C., Maurer M., Grob J.-J., Baskan E.B., Bradley M.S., Canvin J., Rahmaoui A., Georgiou P., Alpan O. (2015). Efficacy and safety of omalizumab in patients with chronic idiopathic/spontaneous urticaria who remain symptomatic on H1 antihistamines: A randomized, placebo-controlled study. J. Investig. Dermatol..

[25] Kaplan A., Ledford D., Ashby M., Canvin J., Zazzali J.L., Conner E., Veith J., Kamath N., Staubach P., Jakob T. (2013). Omalizumab in patients with symptomatic chronic idiopathic/spontaneous urticaria despite standard combination therapy. J. Allergy Clin. Immunol..

[26] Maurer M., Casale T.B., Saini S.S., Ben-Shoshan M., Giménez-Arnau A.M., Bernstein J.A., Yagami A., Stjepanovic A., Radin A., Staudinger H.W. (2024). Dupilumab in patients with chronic spontaneous urticaria (LIBERTY-CSU CUPID): Two randomized, double-blind, placebo-controlled, phase 3 trials. J. Allergy Clin. Immunol..

[27] Casale T.B., Saini S.S., Ben-Shoshan M., Giménez-Arnau A.M., Bernstein J.A., Hayama K., Amin N., Robinson L.B., Bauer D., Dakin P. (2025). Dupilumab improves itch and urticaria activity in patients with chronic spontaneous urticaria: Pooled results from two phase 3 trials (LIBERTY-CSU CUPID Study A and Study C). J. Allergy Clin. Immunol..

[28] Metz M., Giménez-Arnau A., Hide M., Lebwohl M., Mosnaim G., Saini S., Sussman G., Szalewski R., Haemmerle S., Lheritier K. (2025). Remibrutinib in chronic spontaneous urticaria. N. Engl. J. Med..

[29] Maurer M., Giménez-Arnau A., Metz M., Jain V., Stull D., Aldridge S., Heath-Chiozzi M., Ma R., Paradise E., Hawthorne T. (2025). Anti-KIT barzolvolimab for chronic spontaneous urticaria. Allergy.

[30] Khan A.A., Riaz A.A., Naseer F., Fatima N., Abrar Z., Malik L., Khan J., Aslam R., Rab A.A., Khan A. (2025). Efficacy, safety, and quality-of-life outcomes of remibrutinib in chronic spontaneous urticaria: A systematic review and meta-analysis. J. Allergy Clin. Immunol. Pract..

[31] Türk M., Yılmaz İ., Ertaş R., Kocatürk E. (2025). Efficacy of omalizumab, dupilumab, and remibrutinib in chronic spontaneous urticaria: Phase data and placebo response. Allergy.

[32] Xiong G., Rayner D.G., Kim L., Sussman G., Chu D.K. (2025). Comparative efficacy of omalizumab, dupilumab, and remibrutinib in chronic spontaneous urticaria: A network meta-analysis of randomized controlled trials. J. Dermatolog. Treat..

[33] Bernstein J.A., Maurer M., Saini S.S., Griswold N., Mast J., Martzloff E.-D., Rodrigues J., Hawkes J.E. (2026). Efficacy of remibrutinib versus dupilumab at early timepoints in chronic spontaneous urticaria: US phase 3b study design (RECLAIM, NCT06868212). Dermatol. Ther..

[34] Yao X., Liu Y., Luo Y., Liu X., Zhou M., Yang B., Liu L., Li L., Liu W., Man X. (2026). Safety and efficacy of the selective tyrosine kinase 2/Janus kinase 1 inhibitor TLL-018 in patients with moderate-to-severe chronic spontaneous urticaria with inadequate response to H1 antihistamines: A phase Ib, randomized, double-blind, placebo-controlled pilot study. Br. J. Dermatol..

[35] Zhu Y., Zhang W., Wang D., Chen S., Shao Y., Peng L., Shen Y., Tang H. (2026). Real-world outcomes and tolerability of Janus kinase inhibitors in chronic spontaneous urticaria and chronic inducible urticaria: A single-center retrospective study. J. Am. Acad. Dermatol..

[36] Vahabi S.M., Heidari S., Pourgholi E., Memari H., Hashemnejad M.A., Ansari M.S., Esmaeili F., Sadeghi B., Soveizi S., Etesami I. (2026). Janus kinase inhibitors for the treatment of prurigo nodularis: A systematic review of 211 patients. J. Dermatol..

[37] Zhu C.K., Bendayan E., Farzad A., Ben-Shoshan M., Fein M., Netchiporouk E. (2026). Janus kinase inhibitors as a therapeutic option for chronic spontaneous urticaria refractory to antihistamines and omalizumab: A case series. J. Cutan. Med. Surg..

